# Successful Treatment of Disseminated Bacillus Calmette-Guérin Disease in an HIV-Infected Child with a Linezolid-Containing Regimen

**DOI:** 10.1155/2016/1528981

**Published:** 2016-10-10

**Authors:** Srđan Roglić, Drusia Dickson, Branko Miše, Klaudija Višković, Vera Katalinić-Janković, George Rutherford, Josip Begovac

**Affiliations:** ^1^University Hospital for Infectious Diseases, Zagreb, Croatia; ^2^University of California, San Francisco, School of Medicine, San Francisco, CA, USA; ^3^School of Medicine, University of Zagreb, Zagreb, Croatia; ^4^Croatian Institute of Public Health, Zagreb, Croatia

## Abstract

Upon HIV infection diagnosis, an 8-month-old boy was transferred for evaluation of worsening respiratory distress requiring mechanical ventilation.* Pneumocystis jirovecii* pneumonia (PCP) was diagnosed; the boy also had a nonhealing ulcer at the site of vaccination with Statens Serum Institut (Danish strain) Bacillus Calmette-Guérin (BCG) vaccine and associated axillary lymphadenopathy. PCP treatment resulted in weaning from mechanical ventilation. Antimycobacterial treatment was immediately attempted but was discontinued because of hepatotoxicity. Over several months, he developed splenic lesions and then disseminated skin and cystic bone lesions.* M. bovis* was repeatedly cultured from both skin and bone lesions despite various multidrug antimycobacterial regimens which included linezolid. Eventually, treatment with a regimen of rifabutin, isoniazid, ethambutol, and linezolid led to definitive cure. Clinicians should consider a linezolid-containing regimen for treatment of severe disseminated BCG infection, especially if other drug regimens have failed. Although drug toxicity is a particular concern for young children, this patient received linezolid for 13 months without serious toxicity. This case also highlights the need for universal screening among pregnant women to prevent vertical transmission of HIV. Finally, routine immunization with BCG vaccine at birth should be questioned in countries with low and declining burden of tuberculosis.

## 1. Introduction

Disseminated Bacillus Calmette-Guérin (BCG) infection after vaccination has a high mortality rate and occurs mostly in patients with immunologic deficiency [1]. Infants infected with human immunodeficiency virus (HIV) are particularly susceptible [2]. In Croatia, children receive routine BCG vaccine at birth (99% coverage) [3], but it is contraindicated in HIV-exposed infants. However, there is no routine HIV testing of pregnant women in Croatia as vertical HIV transmission is rare and only 14 children have been perinatally infected since 1985 [4]. We report a case of disseminated BCG infection with cystic bone lesions in an HIV-infected child. The child was successfully cured after long-term therapy with an antimycobacterial regimen including rifabutin and linezolid.

## 2. Case Presentation

After HIV infection was diagnosed, an 8-month-old boy with worsening pneumonia and respiratory distress was transferred to the University Hospital for Infectious Diseases, Zagreb, Croatia (UHID), on November 4, 2011. The child had previously been hospitalized two times in his local hospital for diarrhoea and pneumonia. His mother had not been tested for HIV while pregnant. There was no family history of tuberculosis (TB), but immunization with Statens Serum Institut (Danish strain) BCG vaccine at birth had left a nonhealing ulcer on the deltoid region of the left arm.

On presentation, the infant was receiving mechanical ventilation and was overall in poor condition. He weighed 7.4 kg and was 42.5 cm long. On physical examination he had tachycardia, hepatosplenomegaly, and oedema of the lower extremities. There was also an ulcer at the site of BCG immunization with a palpable ipsilateral 1 cm axillary lymph node. His chest X-ray showed diffuse bilateral interstitial infiltrates with a slightly enlarged cardiac silhouette.

**Figure 1:**
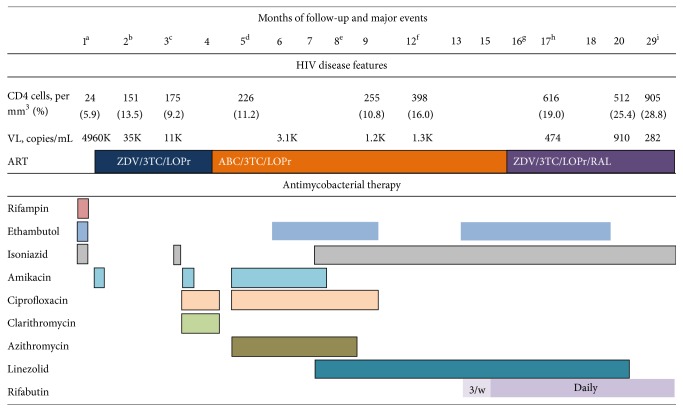
Major events, CD4 cell count and viral load measurements, antiretroviral therapy, and antimycobacterial therapy during the course of the disease. (a)* Pneumocystis jiroveci* pneumonia; culture of tracheal aspirate* M. bovis* positive; liver failure, antituberculosis therapy stopped; QuantiFERON TB test negative; ART started. (b) Hypoechogenic splenic lesions seen on ultrasound. (c) Necrotic lymph nodes seen on ultrasound; urticarial rash, after isoniazid reintroduction. (d) Disseminated skin lesions (*M. bovis* cultured); QuantiFERON TB test positive; blood culture for* M. bovis* negative; cystic bone lesions. (e) Lopinavir/ritonavir replaced by nevirapine for 2 weeks. (f) Tibial bone biopsy culture for* M. bovis* positive. (g) Leg abscess culture for* M. bovis* positive. (h) Skin lesion and lymph node culture for* M. bovis* negative. (i) Antimycobacterial therapy stopped. VL, viral load (HIV-1 RNA); ART, antiretroviral therapy; ZDV, zidovudine; 3TC, lamivudine; LOPr, lopinavir/ritonavir; ABC, abacavir; RAL, raltegravir; 3/w, three times weekly; TB, tuberculosis.

Results of laboratory studies on admission showed an erythrocyte sedimentation rate of 5 mm per first hour, serum procalcitonin level of 0.844 *µ*g/L, hemoglobin concentration of 130 g/L, leukocyte count of 10.4 × 10^9^/L, and platelet count of 51 × 10^9^/L. His CD4 cell count was 5.9% (24 cells per *µ*L) and his plasma HIV-1 RNA was 4.96 million copies/mL. An overview of the major clinical and laboratory findings is presented on Figure 1.

He was immediately given high-dose co-trimoxazole and steroids for presumed* Pneumocystis jirovecii* pneumonia as well as piperacillin-tazobactam for probable hospital acquired infection. Anti-TB therapy consisting of isoniazid, ethambutol, and rifampicin, which had been started the day before transfer to our hospital, was continued. On the third hospital day at UHID he developed signs of hepatitis; his serum bilirubin climbed to 116.5 *µ*mol/L (conjugated bilirubin 66.9 *µ*mol/L), his aspartate aminotransferase (AST) was 1952 IU/L, his alanine aminotransferase (ALT) was 365 IU/L, and his lactate dehydrogenase level was 2948 IU/L. His prothrombin time was prolonged (international normalized ratio value 2.7). All TB medications were stopped and liver function tests gradually returned to normal over the next three weeks.

Tracheal aspirate taken on the day of admission was positive for* Pneumocystis jirovecii* DNA on polymerase chain reaction and also grew* Pseudomonas aeruginosa* on culture. Antiretroviral therapy (ART) was started two weeks after admission to UHID with a combination of zidovudine, lamivudine, and lopinavir/ritonavir. There was no HIV drug resistance found in the blood sample obtained prior to initiation of ART. Steroids were stopped after 21 days of therapy and at that point co-trimoxazole was switched to a prophylactic oral dose. The breathing had significantly improved so he was weaned from mechanical ventilation 4 weeks after admission to our hospital. We performed an interferon-gamma release assay (QuantiFERON®); the result was negative. In light of the clinical improvement and prior signs of severe, presumably toxic hepatitis, the decision was made not to give TB treatment.

Two months after admission an abdominal ultrasound revealed multiple small (up to 8 mm) hypoechogenic splenic lesions. Two weeks later signs of BCG infection were again present with prominent ipsilateral axillary lymph node enlargement. An attempt was made to restart anti-TB medications. However, an urticarial rash developed after isoniazid was started, so the drug was discontinued; the patient was treated with amikacin for one week and clarithromycin, ciprofloxacin, and prednisolone for 4 weeks. This treatment regimen led to regression in lymph node size. At this time, zidovudine was replaced by abacavir due to anaemia (Figure 1).

In the fourth month of care, the child developed multiple skin lesions on his head and limbs that started as erythematous papules and turned into subcutaneous abscesses. A repeated QuantiFERON test was now positive. Mycobacterial cultures from the disseminated skin lesions were taken and subsequently grew* M. bovis*. At this point we learned that mycobacterial cultures from the site of BCG application (left deltoid muscle) and tracheal aspirate were taken at the local hospital about one week prior to transfer to our hospital. These cultures also grew* M. bovis*, but the staff at UHID was not aware of the positive results. Initially the samples were cultured on solid (Loewenstein and Stonebrink) and liquid MGIT (Mycobacteria Growth Indicator Tube, the BD BACTEC*™* MGIT*™* 960 System) media. The identification of the cultured* M. bovis* BCG strain was done using the commercially available line probe assay, GenoType MTBC Kit (Hain Lifescience, Nehren, Germany).

**Figure 2:**
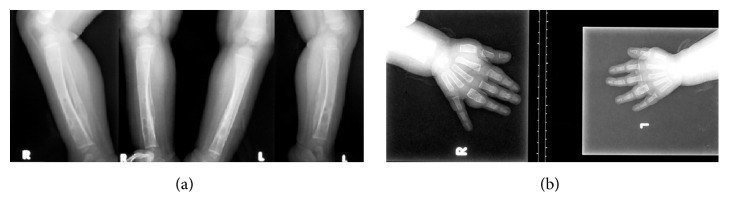
Disseminated Bacillus Calmette-Guérin (BCG) infection of the upper and lower extremities in an infant produces similar images (June–September 2012). (a) Bilateral lateral and anteroposterior conventional radiographs of the lower parts of both legs show multiple radiolucent cystic lytic areas of bone destruction with minimal periosteal reaction and bone widening in the distal diaphysis of tibia and fibula. (b) Conventional posteroanterior radiographs of both hands revealed periosteal elevation and multiple small lytic lesions with bone widening in the proximal phalanges of the fourth fingers bilaterally.

Drugs with antimycobacterial activity (amikacin, ciprofloxacin, and azithromycin) were reintroduced (Figure 1). Multiple cystic bone lesions were noted in the skull and in the bones of both arms, both hands, and both legs (Figure 2). As fever and skin lesions persisted, ethambutol, linezolid (10–12 mg/kg orally three times per day), and isoniazid were sequentially added to his anti-TB regimen (Figure 1). This time neither rash nor toxic hepatitis developed. Because we wanted to give rifampicin again and rifabutin was not available at that time, we replaced lopinavir/ritonavir with nevirapine. However, two weeks later there was an increase in serum liver enzymes (AST 113 IU/L and ALT 122 IU/L), so nevirapine was stopped and standard dose lopinavir/ritonavir was reintroduced. Amikacin, azithromycin, ciprofloxacin, and ethambutol were discontinued as shown in Figure 1. After a 10-month hospital stay the patient was discharged on linezolid, isoniazid, abacavir, lamivudine, and lopinavir/ritonavir.

However, fever reappeared, new skin lesions were still developing, and bone lesions were growing. A bone biopsy of the right tibia was done on 12th month of care at UHID (Figure 1). Histopathological findings revealed inflammatory tissue with microscopic characteristics of granulomata and foci of necrosis, which grew acid-fast bacilli identified as* M. bovis*. At this point ethambutol and rifabutin (dosed at 5 mg/kg three times per week) were added to the TB regimen, and after six weeks the frequency of rifabutin dosing was increased to daily administration (once daily 7 days per week) (Figure 1). After rifabutin was given dosed at 5 mg/kg/day every day the patient remained without fever. The child's complete blood count was monitored; after rifabutin was given 3 times weekly his lowest observed leukocyte count was 4.1 × 10^9^/L with an absolute neutrophil count (ANC) of 1.3 × 10^9^/L (1300 per *µ*L) on the 18th day of administration. After rifabutin was given daily, the lowest leukocyte count was observed after one month of therapy (1.9 × 10^9^/L with an ANC of 0.7 × 10^9^/L (700 per *µ*L)). However, this neutropenia was transient; after six days the ANC was 3300 per *µ*L without any intervention. Since the response to ART was not optimal (Figure 1), a resistance test was done which showed reverse transcriptase mutations associated with nevirapine (Y181C) and lamivudine (M184V) resistance (16th month of care). To optimize the virological response to ART, abacavir was replaced by zidovudine and raltegravir granules were added to the antiretroviral regimen. After 16 months in care a skin lesion and a lymph node aspirate failed to grow mycobacteria for the first time, and one month later the child became consistently afebrile. At this point ethambutol treatment had reached 6 months, and it was discontinued. Two months later linezolid was stopped after a total of 13 months of therapy. At follow-up 12 months from the first negative skin lesion culture the child was clinically stable and radiographs of the lower limb showed signs of bone healing. Anti-TB therapy was stopped at this time (Figure 1). The child reached an undetectable HIV-1 RNA viral load measured by the Abbott assay (detection limit 40 copies/mL) only after 36 months of ART on a regimen of tenofovir, lamivudine, lopinavir/ritonavir, and raltegravir.

## 3. Discussion

Our case report demonstrates the range of difficulties and challenges in the management of disseminated* M. bovis* infection in an HIV-infected infant. Other opportunistic infections can be present as evidenced by the severe, life-threatening* Pneumocystis jirovecii* pneumonia in our patient. Drug toxicities from first line anti-TB drugs occurred, and less effective regimens needed to be given. Nonetheless, despite these toxicities every attempt should be made to reintroduce the most potent anti-TB drugs. Pharmacotherapy is complicated in HIV-infected infants and small children because of the limited number of antiretroviral drug formulations, drug interactions with rifampicin, and drug toxicities. There is also little clinical experience in children with the dosing of rifabutin when combined with lopinavir/ritonavir. A pharmacokinetic study in children without tuberculosis published in 2015 showed that rifabutin dosed at 5 mg/kg three times per week resulted in lower exposure values to rifabutin compared to adults receiving 150 mg of rifabutin daily [5]. In our child, after the dosing of rifabutin was increased to 5 mg/kg daily 7 days per week no recurrencies of fever were observed. So, our experience would suggest that daily administration of rifabutin at 5 mg/kg might be appropriate. Similarly to the study by Moultrie et al. [5], we observed neutropenia during rifabutin therapy; however, it was transient and did not require discontinuation of the drug or a change in dosing frequency. Instead of rifabutin our child could have received “super-boosted lopinavir/ritonavir” with rifampicin. In a pharmacokinetic study, children treated with ritonavir in addition to doses of coformulated lopinavir/ritonavir (super-boosted lopinavir/ritonavir) while on rifampicin-based treatment achieved serum concentrations of lopinavir comparable to those of children treated with standard dose lopinavir/ritonavir in the absence of rifampicin [6]. Based on retrospective clinical observations, virologic response among children receiving super-boosted lopinavir and rifampicin appeared to be similar to that of children receiving standard dose lopinavir/ritonavir without tuberculosis treatment [7]. In our child, super-boosted lopinavir/ritonavir was not given together with rifampicin mainly because ritonavir in the liquid form was not available in Croatia. Because our patient experienced hepatotoxicity when given rifampicin and later also on nevirapine, we were also concerned that hepatotoxicity might develop if rifampicin was given with super-boosted lopinavir/ritonavir.

ART-induced immune recovery may unmask or worsen existing* M. bovis* infection. As in our example, it is sometimes difficult to judge whether the worsening and persistence of the disease represent an uncontrolled active infection or an immune reconstitution syndrome.

Children in Croatia are routinely immunized with an attenuated BCG strain of* Mycobacterium bovis* at birth to prevent tuberculosis. Disseminated BCG infection is the most serious complication of BCG vaccination, with an incidence around one in one million vaccine recipients and mortality rate over 50% in those that develop serious complications [8, 9]. It almost always occurs in patients with immunodeficiency disorders, especially cell-mediated immune defects such as severe combined immunodeficiency, chronic granulomatous disease, and HIV infection [1]. Our patient presented with relapsing fever, failure to thrive, and hepatosplenomegaly, which are among the most commonly reported symptoms of disseminated BCG infection. He also developed multiple skin abscesses and, later in the course of disease, multiple bone lesions.* M. bovis* was repeatedly cultured from both skin and bone lesions and showed the usual susceptibility pattern of being resistant only to pyrazinamide. This patient had lesions in the long bones of the extremities, as commonly seen in disseminated infection, but also had skull lesions, which is not a common finding [10]. New skin lesions appeared despite combined anti-TB treatment until first line anti-TB drugs were reintroduced. Although disseminated BCG infection (including cases with multiple bone lesions) are often seen in children with severe combined immunodeficiency, reports of similar cases in HIV-infected children are scarce [11–13].

According to the revised pediatric classification of BCG disease proposed by Hesseling et al. [11], our patient had local skin disease that progressed to distant disease (pulmonary involvement) and then disseminated disease (skin and bone involvement). The disseminated disease unfolded after a rise in CD4 cell count and a drop in HIV-1 RNA viral load suggesting a role for immune reconstitution. The first appearance of extrapulmonary BCG infection (hypoechogenic splenic lesions) was noted 8 weeks after starting ART and coincided with an increase in the CD4 lymphocyte cell count. However, one may argue that the worsening of symptoms was primary due to the inadequate TB treatment and not to immune reconstitution. The interferon-gamma release assay (QuantiFERON) is believed not to be affected by* M. bovis* infection. However, the positive test at the time of immune recovery suggests that a false positive test may occur in some circumstances. Cases of BCG-induced immune reconstitution syndrome have been described in HIV-infected children, but all reported cases in the English medical literature describe only localized suppurative disease [14, 15].

Linezolid is currently used mainly for the treatment of infections caused by resistant Gram-positive bacteria. Although the use of linezolid for mycobacterial infections is increasing, the World Health Organization categorizes linezolid as group 5 anti-TB agent with unclear efficacy or concerns regarding usage [16]. Linezolid has shown* in vitro* activity against* Mycobacterium tuberculosis* [17]. Another study reported similar linezolid MIC values of* M. bovis* and* M. tuberculosis* isolates (0.5 mg/L), suggesting susceptibility to linezolid [18].* In vivo* studies described dose-dependent activity of linezolid in a murine model of TB and limited bactericidal activity in mice [19, 20]. One* in vitro* study showed that linezolid had synergistic activity with rifampicin but not with the fluoroquinolones in* M. tuberculosis* infection [21], while multiple studies in mice reported antagonistic activity with isoniazid, rifampicin, and pyrazinamide [22]. However, an increasing number of case reports and studies present good results with linezolid-containing regimens for the treatment of drug-resistant* M. tuberculosis* infection in both adults and children [23–27]. Our patient improved clinically after linezolid was added to the treatment regimen, but he still had periods of low-grade fever and developed new skin and bone lesions. The addition of rifabutin to the anti-TB regimen eventually resulted in complete cure of the infection. Although linezolid has a good safety profile, long-term treatment increases the likelihood of side-effects, especially bone marrow suppression, which sometimes requires cessation of therapy [28]. Other serious side-effects are optic and peripheral neuropathy. Our patient received linezolid for more than a year and required red blood cell transfusions three times during linezolid therapy. However, because of the severity of the disease and other concomitant medications (co-trimoxazole), it is unlikely that linezolid was the main cause of anaemia. Moreover, blood transfusions were given early during linezolid therapy; when co-trimoxazole was stopped there was a significant improvement in red blood cell count with no further need for additional blood transfusions despite continued treatment with linezolid.

Universal BCG vaccination is common in the world; 157 countries recommended it in 2010 [3]. However, given the constantly declining rates of TB in Croatia, [29] it may be appropriate to abandon universal BCG vaccination of newborns in Croatia as well as in other countries with declining and low TB incidence. To avoid cases of disseminated BCG disease caused by vaccination of newborns, it is important to know the HIV status of the mother and the newborn. Had HIV infection in the mother been diagnosed during pregnancy, the BCG vaccine would not have been given until HIV infection in the newborn was ruled out and vertical HIV transmission to the child could have also been prevented by antiretroviral treatment and prophylaxis. Our case highlights the need for universal HIV screening among pregnant women even in settings with a low-level HIV epidemic.

## Abbreviations

BCG: Bacillus Calmette-Guérin
HIV: Human immunodeficiency virus
UHID: University Hospital for Infectious Diseases, Zagreb, Croatia
TB: Tuberculosis
AST: Aspartate aminotransferase
ALT: Alanine aminotransferase
ART: Antiretroviral therapy
ANC: Absolute neutrophil count.
